# Obesity partially potentiates dimethylbenz[a]anthracene-exposed ovotoxicity by altering the DNA damage repair response in mice^†^

**DOI:** 10.1093/biolre/ioac218

**Published:** 2023-01-25

**Authors:** Jaspreet K Rishi, Kelsey Timme, Hunter E White, Karl C Kerns, Aileen F Keating

**Affiliations:** Department of Animal Science, Iowa State University, Ames, IA, USA; Department of Animal Science, Iowa State University, Ames, IA, USA; Department of Animal Science, Iowa State University, Ames, IA, USA; Department of Animal Science, Iowa State University, Ames, IA, USA; Department of Animal Science, Iowa State University, Ames, IA, USA

**Keywords:** ovary, obesity, DMBA, DNA repair, BRCA1

## Abstract

Obesity adversely affects reproduction, impairing oocyte quality, fecundity, conception, and implantation. The ovotoxicant, dimethylbenz[a]anthracene, is biotransformed into a genotoxic metabolite to which the ovary responds by activating the ataxia telangiectasia mutated DNA repair pathway. Basal ovarian DNA damage coupled with a blunted response to genotoxicant exposure occurs in obese females, leading to the hypothesis that obesity potentiates ovotoxicity through ineffective DNA damage repair. Female KK.Cg-a/a (lean) and KK.Cg-Ay/J (obese) mice received corn oil or dimethylbenz[a]anthracene (1 mg/kg) at 9 weeks of age for 7 days via intraperitoneal injection (*n* = 10/treatment). Obesity increased liver weight (*P* < 0.001) and reduced (*P* < 0.05) primary, preantral, and corpora lutea number. In lean mice, dimethylbenz[a]anthracene exposure tended (*P* < 0.1) to increase proestrus duration and reduced (*P* = 0.07) primordial follicle number. Dimethylbenz[a]anthracene exposure decreased (*P* < 0.05) uterine weight and increased (*P* < 0.05) primary follicle number in obese mice. Total ovarian abundance of BRCA1, γH2AX, H3K4me, H4K5ac, H4K12ac, and H4K16ac (*P* > 0.05) was unchanged by obesity or dimethylbenz[a]anthracene exposure. Immunofluorescence staining demonstrated decreased (*P* < 0.05) abundance of γH2AX foci in antral follicles of obese mice. In primary follicle oocytes, BRCA1 protein was reduced (*P* < 0.05) by dimethylbenz[a]anthracene exposure in lean mice. Obesity also decreased (*P* < 0.05) BRCA1 protein in primary follicle oocytes. These findings support both a follicle stage-specific ovarian response to dimethylbenz[a]anthracene exposure and an impact of obesity on this ovarian response.

## Teaser

Different responses to DMBA exposure occur in lean and obese mice, indicating that obesity impairs DNA repair in the ovary.

## Introduction

The female gonads are ubiquitously exposed to environmental insults, which can cause temporary or permanent loss of fertility [1–3]. Since females are born with a finite number of primordial follicles, once this pool is exhausted, loss of ovarian function and permanent cessation of menstruation [4] ensues [5]. The median age of US women at menopause is 50.2 years [6], but this can be accelerated by exposure to xenobiotics [1, 7, 8]. Loss of follicles is also accompanied by a decline in the level of ovarian hormones, predisposing individuals to increased risk of morbidity [9].

Dimethylbenz(a)anthracene (DMBA) is a model ovotoxic polycyclic aromatic hydrocarbon (PAH) which increases atresia in ovarian follicles of all stages [9–11]. The presence of PAHs is detectable in cigarette smoke [12], diesel exhaust, charred meat, overheated cooking oil, petroleum coke or asphalt manufacturing, waste incineration, wildfires [13–15], and they are air pollutants [16]. In humans, routes of PAH exposure are via inhalation and dermal contact [17]. Post-absorption, DMBA is converted into a genotoxic epoxide metabolite via ovarian microsomal epoxide hydrolase (EPHX1) [18] leading to DNA adduct formation [18,19]. Higher levels of ovarian EPHX1 have been detected in obese mice, suggesting greater potential exposure to the ovotoxic DMBA metabolite [20].

Ovarian genotoxicant exposure results in a concomitant increase in the abundance of DNA damage repair (DDR) proteins [21]. Improperly repaired DNA can lead to the loss of genomic integrity, altering cell growth or leading to cell death [21,22]. The ovary is capable of DDR [23] and responds to DNA double-stranded breaks (DSBs) by activating DDR proteins, including those in the ataxia telangiectasia mutated (ATM) pathway [24]. Upon sensing DSBs, ATM triggers a cascade of phosphorylation events, leading to cell cycle arrest for DNA repair, and if the damage is beyond repair, the cell undergoes apoptosis [25]. Phosphorylation of the histone H2AX (γH2AX) by ATM results in binding to DNA and recruitment of DDR proteins, including breast cancer type 1 susceptibility protein (BRCA1) [25]. It has previously been demonstrated that progressive obesity decreases BRCA1 abundance in mouse ovaries [22] and in obese mice, there is dysfunction in ovarian ATM phosphorylation in an ovarian follicle stage dependent manner and a subsequent disconnect between ATM and H2AX phosphorylation [26]. Thus, basal ovarian DNA damage is observed during obesity along with a blunted response to genotoxicant exposure [20,21].

Post-translational modifications of histones can regulate ovarian follicle formation and maturation; H3K4me has roles in follicular development and embryonic viability [27], H4K5ac facilitates DDR due to its chromatin de-condensation properties [28] while H4K12ac and H4K16ac are involved in DDR by preventing RAD5-dependent CAG repeats [29]. Histone modifications reported to be involved in DNA repair include H3K4me [30], H4K5ac [31], H4K12ac [29,31], and H4K16ac [29]. Thus, there is a role for histone modification in the response to DNA damage, though the precise involvement in the ovary is unclear.

Obesity is a global epidemic due to two main causes: increased caloric intake and a sedentary lifestyle [32,33]. This noncommunicable disease is characterized by increased body fat percentage, insulin resistance, and hypertension [32]. Obesity detrimentally affects female fertility [34], lowering oocyte quality [35], decreasing fecundity [36], and increasing risk of birth defects [37]. In a mouse model of hyperphagia, progressive obesity induced inflammation [38], altered steroidogenesis [38], depleted the ovarian reserve [38], altered gap junction communication [39], induced basal DNA damage [21], and altered the ovarian response to ovotoxicants [20–22,39,40]. Specifically, in the ovaries of obese mice, DMBA-induced abundance of DDR [21] and chemical biotransformation [40] proteins was altered relative to lean mice, implying a differential response to ovotoxicant exposure.

The objective of this study was to investigate the ovarian response to DMBA-induced DSB formation at a time prior to an obesity-induced decline in primordial follicle number [38]. The DMBA exposure paradigm used a dose demonstrated to be ovotoxic (1 mg/kg/day) but for 7 days rather than 14 days as performed in previous studies [20,21] to ensure that modes of ovotoxicity could be investigated in small pre-antral follicles prior to their destruction since the longer exposure duration almost completely decimated ovarian follicle numbers. In addition, the impact of obesity on ovotoxicant-induced DDR was investigated. We hypothesized that obesity potentiates ovotoxicity through ineffective DDR, and investigated changes to abundance of DDR proteins, γH2AX, and BRCA1 and their distribution patterns in the ovary in lean and obese mice. Also, histone modifications reported to have DDR roles (H3K4me, H4K5ac, H4K12ac, and H416ac) were quantified in lean and obese mice exposed to DMBA.

## Materials and methods

### Reagents

MINI-PROTEAN TGX gels (4–20%) were obtained from Bio-Rad Laboratories, Inc. (Hercules, CA, USA). 7,12-Dimethylbenz(a)anthracene (CAS # 57–97-6), 2-β-Mercaptoethanol, Ethylenediaminetetraacetic acid (EDTA), eosin Y, glycerol, glycine, hematoxylin, 4-(2-hydroxyethyl)-1-piperazineethanesulfonic acid (HEPES), NaF, nonfat dry milk, paraffin, phosphate-buffered saline (PBS), Tris base, and Tris-buffered saline (TBS) were obtained from Sigma-Aldrich (St. Louis, MO, USA). Enhanced Chemiluminescence detection substrate (ECL) was obtained from SignalFire (San Francisco, CA, USA). Citrasolv; Pierce bicinchoninic acid assay (BCA) kit, Ponceau S stain, Restore PLUS western blot stripping buffer and SlowFade Gold mounting media were obtained from Thermo Fisher Scientific (Rockfield, IL, USA). Protease/phosphatase inhibitor cocktail was purchased from Cell Signaling Technology (catalog # 5872; Danvers, MA, USA).

### Animal exposure and tissue collection

Female C57Bl6 (KK.Cg-a/a; *n* = 20) wild-type mice, designated as HPL (hyperphagic lean) and agouti lethal yellow (KK.Cg-Ay/J; *n* = 20), designated as HPO (hyperphagic obese), were obtained from Jackson Laboratory (Bar Harbor, ME, USA) and housed at Iowa State University, as approved by the Institutional Animal Care and Use Committee. Mice were housed two to five per cage and maintained at a 12-h circadian rhythm and 25°C. Food (2014 Envigo Teklad Global 14% Protein Rodent Maintenance Diet) and water were available ad libitum. Weekly food intake was quantified by averaging the food consumed per mouse per day in each cage, and body weights were monitored. Animals were obtained at 6 weeks of age, and the 7-day DMBA dosing period began at ~9 weeks of age, ensuring acclimatization and at least a 30% weight difference between the HPL and HPO mice. Control (CT) mice received corn oil, whereas the treatment groups received DMBA (1 mg/kg/day) via intraperitoneal injection. This dose of DMBA for 14 days has been determined previously to destroy almost all primordial and primary follicles [20]; thus, a shorter duration of dosing was employed to ensure that primordial and primary were present to study modes of toxicity. Euthanasia occurred on day 2 of diestrus post-dosing, at 10 weeks of age, to ensure lack of variation in the estrous cycle hormonal milieu. Total body weight was recorded at euthanasia, followed by spleen, liver, ovary, and uterus weight. Blood was collected immediately, and serum was collected and stored at −80°C. One ovary from each mouse was frozen in liquid nitrogen and stored at −80°C for protein analysis, and the other was fixed in 4% paraformaldehyde overnight at 4°C and transferred to 70% ethanol and stored at 4°C for histological analyses.

### 17β-Estradiol quantification

A blood sample was collected utilizing cardiac puncture immediately following euthanasia. The blood was allowed to clot at room temperature for 15 min, transferred to ice, and centrifuged at 1500 × g at 4°C for 10 min. Serum was shipped to the Ligand Assay and Analysis Core Library at the University of Virginia for analysis of 17β-estradiol by radioimmunoassay (Calbiotech Mouse/Rat ELISA). The assay range was 3–300 pg/ml, and samples with a CV value >20 were disregarded. The mean interassay CV for the analyzed samples was 6.22%.

### Estrous cyclicity monitoring

Vaginal cytology was performed daily to monitor the estrous cycle through the dosing period (7 days) until day 2 of diestrus post-exposure. Saline solution (0.9%) was flushed in the vaginal opening of each mouse, aspirated three to five times and wet vaginal smears were observed directly under the microscope. The stage of the estrous cycle was determined based on the presence of three cell types, i.e., nucleated epithelial cells, cornified epithelial cells, and leukocytes, as described previously [40]. Proestrus phase is marked by nucleated epithelial cells, while some cornified epithelial and leukocytes may be present. Estrus phase comprises large cornified epithelial with no nucleus. Metestrus is marked by mostly leukocytes and some cornified and nucleated epithelial cells. Lastly, diestrus phase is indicated by predominantly polymorphonuclear leukocytes.

### Protein isolation and western blotting

Protein isolation was performed by lysing ovaries (*n* = 5 per treatment) in buffer comprising 1% Triton-x-100, 50 mM HEPES, 150 mM NaCl, 10% glycerol, 50 mM NaF, 2 mM EDTA, and 0.1% SDS. Protease and phosphatase inhibitors (1%) were freshly added. Tissue samples were homogenized briefly and placed on ice for 30 min. Following lysing, samples were centrifuged twice at 10,000 rpm for 15 min, and the supernatant was collected to quantify total protein by performing BCA. Protein (5 μg) was loaded in separate wells of precast 4–20% MINI-PROTEAN TGX gels, separated by 60 V for 15 min followed by 120 V for 1 h, and transferred onto a nitrocellulose membrane using transfer buffer comprising 25 mM Tris base, 192 mM glycine, and 20% methanol. Blocking buffer, comprising 5% nonfat dried milk with 1X TBST, was used to dilute antibodies (Table 1), and membranes were blocked for 1 h before incubating with primary antibody overnight. The membrane was washed three times for 10 min each in TBS followed by incubation in secondary antibody for 1 h. The membrane was washed again and then incubated in ECL for 7 min and exposed to an X-ray film. ImageJ software (National Institutes of Health) was used to densitometrically quantify correct-sized bands. Protein bands were normalized to total protein in each well which was determined by Ponceau S staining to account for discrepancy in gel loading.

**Table 1:** Dilutions of antibodies used for western blotting

1° Antibody	Description	Dilution	2° Antibody	Dilution
BRCA1	Santa Cruz Biotech-6954	1:100	AM	1:2000
γH2AX	Cell Signaling 2577S	1:100	AR	1:1000
H3K4me	Novus Biologicals 21–1023	1:500	AR	1:5000
H4K5ac	Novus Biologicals 21–2024	1:500	AR	1:10,000
H4K12ac	Novus Biologicals 21–2064	1:500	AR	1:10,000
H4K16ac	Novus Biologicals 21–2077	1:100	AR	1:5000

AM = anti-mouse; AR = anti-rabbit.

### Follicle classification and enumeration

Ovaries (*n* = 5 per treatment) were embedded in paraffin at the Iowa State University Veterinary Medicine Histopathology Laboratory and sectioned at 5 μM thickness. Every sixth section was mounted on a slide, with two sections per slide. Tissue sections were stained with hematoxylin and eosin, and slides were blinded before counting follicles to remove counter bias. Healthy follicles on every 12th section were counted using a Leica DM 500 microscope equipped with ICC50W microscope. Healthy follicles contained an oocyte nucleus whereas unhealthy follicles were distinguishable by demonstration of pyknosis and intense eosinophilic staining. Follicles were classified as primordial, primary, secondary, pre-antral, and antral following the procedures described previously [41,42]. The number of corpora lutea which are oocyte-devoid was averaged across all the sections of each ovary.

### Protein localization and quantification

Ovary sections (*n* = 4 per treatment) from each treatment group were immunofluorescently stained to detect BRCA1, pBRCA1, and γH2AX protein localization and abundance. Ovaries were sectioned in the same manner as for follicle counting. Slides were warmed in a water bath at 60°C for 30 min followed by incubation in Citrasolv (3X for 5 min). Tissues were rehydrated in 100% and 70% ETOH 2X for 3 min each and incubated in PBS for 5 min. Antigen retrieval was performed using Tris base buffer (pH 9) in a water bath between 95°C and 100°C for 30 min. Slides were allowed to cool down for 30 min at room temperature and washed in PBS for 2X for 2 min. Sections were encircled with a hydrophobic PAP pen and blocked with 5% mammalian serum (goat/donkey) for 1 h. This was followed by washing the sections in PBS 3X for 5 min and incubating in primary antibody (Table 2) overnight at 4°C in a humidified chamber. The next steps were performed in a darkened room. Slides were washed in PBS (3X for 5 min) and incubated in the appropriate secondary antibody (Table 2) combined with YOYO-1, a cellular DNA stain, for 1 h at room temperature in a humidified chamber. Slides were washed again in PBS (3X for 5 min), and coverslips were added using SlowFade Gold mounting media after blotting. Images were obtained with a Leica DM6 B microscope outfitted with a Lecia K5 camera at the appropriate wavelength for each stain. Images were acquired using Lecia Application Suite X software, and the intensity of the stains in specific follicle types and compartments was analyzed using ImageJ software. To evaluate the presence of DDR proteins and the impacts of DMBA exposure in lean and obese mice thereon, all follicle stages were evaluated for the presence of DDR proteins with quantification in the follicle stages in which specific proteins were apparent. For γH2AX protein, the number of foci was counted in secondary (*n* = 3 sections from individual mice/treatment) and antral (*n* = 4 sections from individual mice/treatment) follicles. For BRCA1 protein, all healthy positively stained primary follicles (*n* = 5–8 follicles per section from five individual mouse ovaries per treatment) and secondary (*n* = 5–12 follicles per section from five individual mouse ovaries per treatment) were quantified in each ovary by measuring the intensities of the stain within the oocyte using Image J. In addition, pBRCA1 was quantified using Image J in all atretic follicles (*n* = 4 mice per treatment). Both primary-only, species-specific IgG antibody in place of primary antibody and secondary-only antibody staining confirmed specificity for each analyzed protein.

**Table 2:** Dilutions of antibodies used for immunofluorescence staining

1°/2° Antibody	Description	Dilution
BRCA1	Santa Cruz Biotech-6954	1:100
γH2AX,	Novus Biologicals 100-384	1:100
pBRCA1	Thermofisher PA536627	1:100
YOYO-1 Iodide (491/509)	Thermofisher Y3601	1:5000
Goat anti-Mouse IgG, Alexa Fluor 568	Thermofisher A-11004	1:500
Goat anti-Rabbit IgG, Alexa Fluor 568	Thermofisher A-11011	1:500

### Statistical analysis

Comparisons were made between treatments using GraphPad Prism 9.0 using unpaired t-test with Welch’s correction. Two-way ANOVA function was used to analyze an interaction between obesity and DMBA exposure. The error bars reported are the standard error of the mean (SEM). Statistical difference was reported at *P* < 0.05, whereas a tendency for a biologically meaningful statistical difference was considered at *P* < 0.10.

## Results

### DMBA exposure reduces uterine weight in obese mice

**Figure 1:**
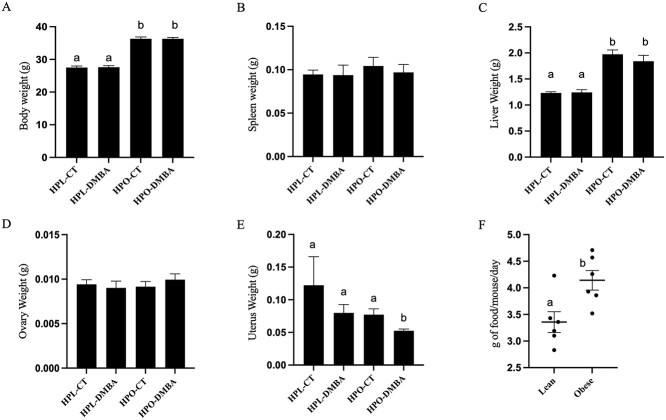
Impact of DMBA exposure on total body and organ weight in lean and hyperphagia obese mice. Following 7 days of exposure to corn oil (CT) or DMBA in lean (HPL) or obese (HPO) mice, weights of (A) body, (B) spleen, (C) liver, (D) ovary, and (E) uterus were recorded. (F) Amount of food consumed by each mouse during the dosing period was also recorded. Different letters indicate differences between treatments; *P* < 0.05; *n* = 10.

Post euthanasia, total body and organ weights were recorded. Obese mice had a higher body weight (Figure 1A; *P* < 0.001) which was induced by hyperphagia as the agouti lethal yellow mice consumed more food per day than the non-agouti lean mice (Figure 1F). Obesity also increased liver weight (Figure 1C; *P* < 0.001). Exposure to DMBA reduced uterine weight in obese but not lean mice (Figure 1E; *P* < 0.05). Spleen and ovary weight were unchanged by obesity or DMBA exposure (Figure 1B and D; *P* > 0.1).

### Exposure to DMBA tended to prolong time spent in proestrus in lean mice

**Figure 2:**
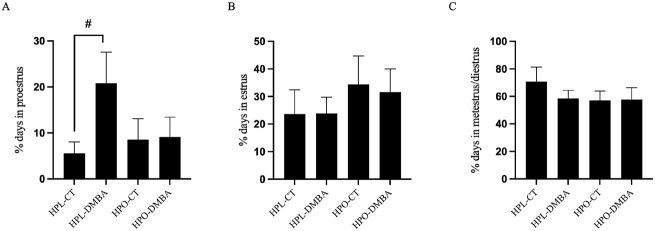
Impact of DMBA exposure on estrous cyclicity of lean and obese mice. The total percentage of days spent in (A) proestrus, (B) estrus, and (C) diestrus + metestrus were recorded during the dosing period (7 days) and until day 2 of diestrus. Data are presented as the percentage of days spent in each phase. # = *P* < 0.10; *n* = 10.

Vaginal cytology was performed daily during the 7-day dosing period and until euthanasia which was determined by the mice being in diestrus for 2 days. There was a tendency (*P* = 0.08) for exposure to DMBA to prolong the percentage of time spent in proestrus in lean mice but not in obese mice (Figure 2A). No additional cyclicity effects were observed at any other stage (Figure 2B and C; *P* > 0.1).

### Obesity affects 17β-estradiol concentration in DMBA-exposed mice

**Figure 3:**
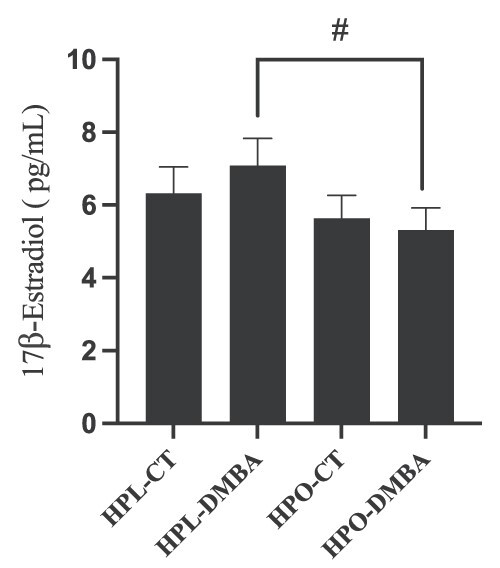
Impact of DMBA exposure on serum 17β-estradiol levels of lean and obese mice. The amount of circulating serum 17β-estradiol was measured via ELISA. Concentrations of estrogen in pg/mL are reported here. # = *P* < 0.1; *n* = 5–10 as some samples were below detection levels.

The amount of circulating serum 17β-estradiol was measured via ELISA. A tendency (*P* = 0.08) for decreased circulating 17β-estradiol levels in DMBA-exposed obese mice as compared with DMBA-exposed lean mice was observed (Figure 3).

### Both obesity and DMBA exposure affect follicle number

**Figure 4:**
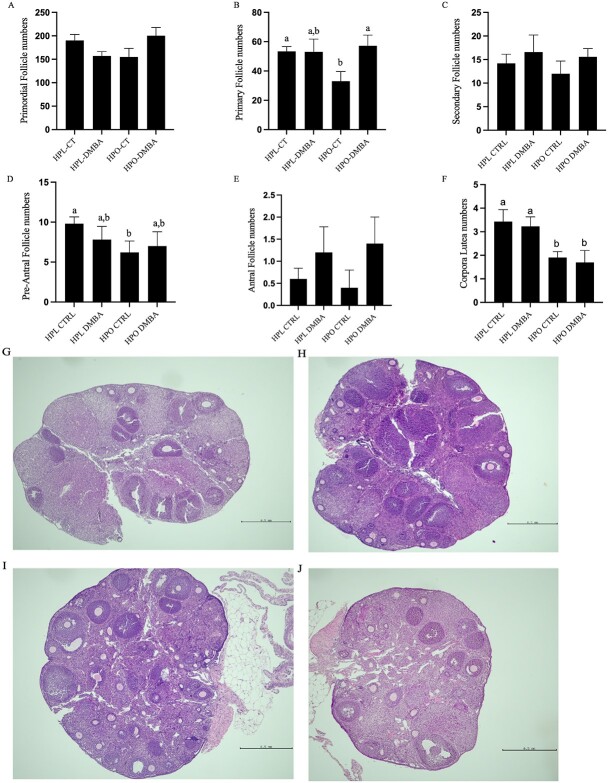
Impact of DMBA exposure on ovarian follicle number in lean and obese mice. Follicles were classified and counted after 7 days of exposure to corn oil (CT) or DMBA in lean or obese mice. Number of (A) primordial follicles (treatment effect *P* = 0.70; obesity effect *P* = 0.80; interaction effect *P* = 0.0180), (B) primary follicles (treatment effect *P* = 0.09; obesity effect *P* = 0.25; interaction effect *P* = 0.09), (C) secondary follicles (treatment effect *P* = 0.27; obesity effect *P* = 0.55; interaction effect *P* = 0.83), (D) pre-antral follicles (treatment effect *P* = 0.69; obesity effect *P* = 0.16; interaction effect *P* = 0.35), (E) antral follicles (treatment effect *P* = 0.11; obesity effect *P* > 0.99; interaction effect *P* = 0.68), and (F) corpora lutea were recorded (treatment effect *P* = 0.65; obesity effect *P* > 0.0027; interaction effect *P* = 0.99). Representative ovary sections stained by hematoxylin and eosin for each treatment group (G) HPL-CT, (H) HPL-DMBA, (I) HPO-CT, and (J) HPO-DMBA are shown. Scale bar = 0.5 mm. Different letters indicate differences between treatments; *P* < 0.05; *n* = 5.

Healthy follicles at different developmental stages were counted. Relative to lean mice, obesity reduced numbers of primary (Figure 4B), pre-antral follicles (Figure 4D), and corpora lutea (Figure 4F; *P <* 0.05). DMBA tended (*P =* 0.07) to decrease primordial follicle number in lean mice, while the number of primordial follicles in DMBA exposed obese mice tended to be higher (*P =* 0.07) than in lean control treated mice (Figure 4A). Relative to CT-treated mice, there were higher numbers of primary follicles in DMBA-exposed obese mice (Figure 4B), but this was not observed in lean mice. It should be noted that the number of primary follicles in CT-treated obese mice was lower (*P* < 0.05) than that of the CT-treated lean mice. There was no effect of DMBA exposure in lean or obese mice on secondary (Figure 4C), pre-antral (Figure 4D), antral (Figure 4E), or corpora lutea (Figure 4F) (*P* > 0.05). As with primary follicle number, CT-treated obese mice had lower numbers (*P* < 0.05) of pre-antral follicles and corpora lutea than identically treated lean mice. An interaction effect between obesity and DMBA for primordial follicle number (*P* = 0.018) was noted.

### D‌DR and histone modifications proteins are unaffected by DMBA and obesity

**Figure 5:**
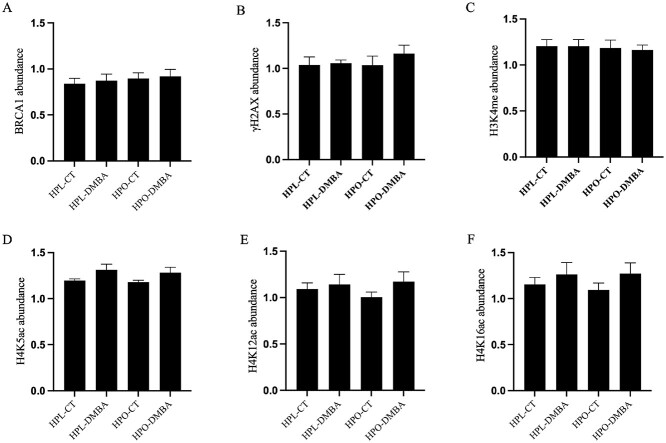
Impact of DMBA on ovarian DNA damage repair and histone modification proteins in lean and obese mice. Following 7 days of exposure to vehicle control (CT) or DMBA, (A) BRCA1 (treatment effect *P* = 0.70; obesity effect *P* = 0.45; interaction effect *P* = 0.95), (B) γH2AX (treatment effect *P* = 0.39; obesity effect *P* = 0.55; interaction effect *P* = 0.52), (C) H3K4me (treatment effect *P* = 0.87; obesity effect *P* = 0.68; interaction effect *P* = 0.87), (D) H4K5ac (treatment effect *P* = 0.03; obesity effect *P* = 0.62; interaction effect *P* = 0.87), (E) H4K12ac (treatment effect *P* = 0.23; obesity effect *P* = 0.75; interaction effect *P* = 0.50), and (F) H4K16ac (treatment effect *P* = 0.18; obesity effect *P* = 0.80; interaction effect *P* = 0.74) abundances were measured through western blot. *P* > 0.05; *n* = 5 ovaries from individual mice per treatment.

The total ovarian abundance of DNA damage repair proteins, BRCA1 and γH2AX, and histone modification proteins, H3K4me, H4K5ac, H4K12ac, and H4K16ac, was measured by western blotting, and all were unchanged in lean and obese mice exposed to DMBA (Figure 5A–F; *P* > 0.05). Additionally, obesity alone did not impact the abundance of BRCA1, γH2AX, H3K4me, H4K5ac, H4K12ac, or H4K16ac (Figure 5A–F; *P* > 0.05). However, a treatment effect on the abundance of H4K5ac was observed (Figure 5D; *P* < 0.05).

### Obesity decreases the abundance of γH2AX in antral follicles

**Figure 6:**
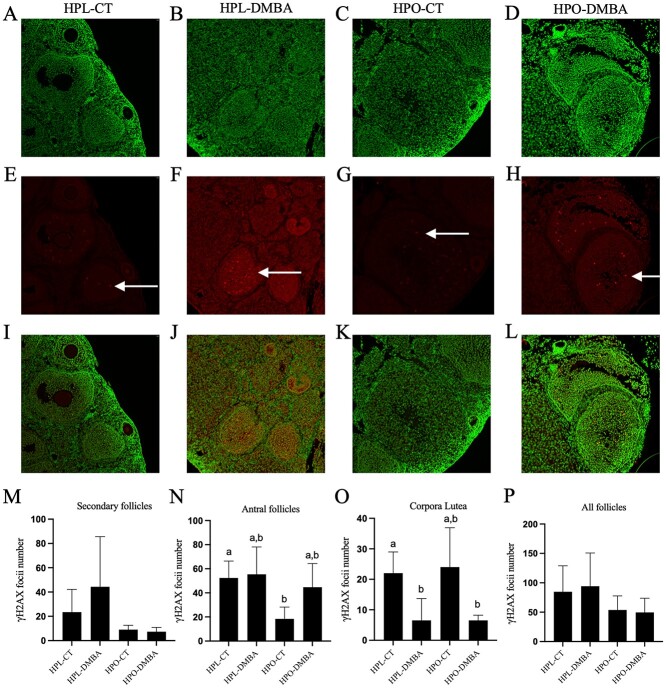
Impact of DMBA on ovarian γH2AX localization in lean and obese mice. Following 7 days of exposure to vehicle control (CT) or DMBA, healthy follicles immunostained to detect γH2AX protein (red) in (A), (E), (I) HPL-CT; (B), (F), (J) HPL-DMBA; (C), (G), (K) HPO-CT; and (D), (H), (L) HPO-DMBA were analyzed. Cellular DNA is stained in green. γH2AX abundance was quantified by counting γH2AX foci (indicated by arrows in F-H) in (M) secondary follicles (treatment effect *P* = 0.48; obesity effect *P* = 0.08; interaction effect *P* = 0.41), (N) antral follicles (treatment effect *P* = 0.18; obesity effect *P* = 0.06; interaction effect *P* = 0.28, (O) corpora lutea (treatment effect *P* = 0.0017; obesity effect *P* = 0.82; interaction effect *P* = 0.82), and (P) all growing follicles (treatment effect *P* = 0.90; obesity effect *P* = 0.08; interaction effect *P* = 0.73). Images were captured at 20× magnification. Different letters indicate differences between treatments; *P* < 0.05; *n* = 4 ovaries from individual mice per treatment.

The localization of γH2AX was assessed in different follicle stages as measured through immunofluorescence staining and quantified by counting the number of γH2AX-stained foci (Figure 6A–P). Obesity decreased the abundance of γH2AX foci in antral follicles relative to lean control treated mice (Figure 6N; *P* < 0.05). In corpora lutea, DMBA exposure decreased γH2AX foci in lean mice (*P* < 0.05), whereas in obese mice, DMBA exposure tended to decrease γH2AX foci (Figure 6O; *P* < 0.07). When γH2AX stained foci in all follicle types were combined, no effects of obesity or DMBA exposure were observed (*P* > 0.1).

### Obesity and DMBA both reduce the abundance of BRCA1 in the oocytes of primary follicles

**Figure 7:**
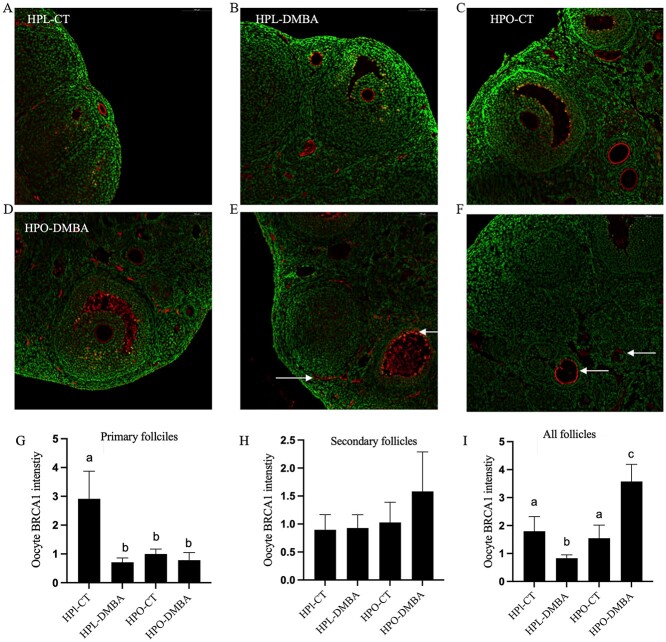
Impact of DMBA on oocyte BRCA1 localization in lean and obese mice. Following 7 days of exposure to vehicle control (CT) or DMBA, oocyte membranes of healthy follicles immunostained by BRCA1 (red) in (A) HPL-CT, (B) HPL-DMBA, (C) HPO-CT, and (D) HPO-DMBA were observed. Positive BRCA1 staining was also observed in (E) thecal cells and antral fluid and (F) atretic follicles (indicated by arrows). Cellular DNA is stained in green. Intensities of the BRCA1 stain were quantified in oocytes of (G) primary follicles (treatment effect *P* = 0.02; obesity effect *P* = 0.06; interaction effect *P* = 0.04), (H) secondary follicles (treatment effect *P* = 0.50; obesity effect *P* = 0.37; interaction effect *P* = 0.54), and (I) all follicles combined (treatment effect *P* = 0.22; obesity effect *P* = 0.005; interaction effect *P* = 0.0009), and the averages from each treatment are reported here. Images were captured at 20× magnification. Different letters indicate differences between treatments; *P* < 0.05; *n* = 4 ovaries from individual mice per treatment.

The localization of oocyte BRCA1 was quantified in primary and secondary follicles as measured through immunofluorescence staining and analyzed using ImageJ software. BRCA1 localization was observed in the oocyte membrane in all treatment groups (Figure 7A–D), thecal cells, antral fluid (Figure 7E), and atretic follicles (Figure 7F). In primary follicles, both DMBA and obesity reduced the abundance of oocyte BRCA1 (Figure 7G; *P* < 0.05) but this was not observed in secondary follicles (Figure 7H). When the abundance of BRCA1 was combined from all follicles (primary, secondary, and antral), DMBA exposure reduced oocyte BRCA1 in lean mice but increased oocyte BRCA1 in obese mice (Figure 7I; *P* < 0.05).

### No observable effects on the distribution of pBRCA1 when exposed to DMBA or obesity

**Figure 8:**
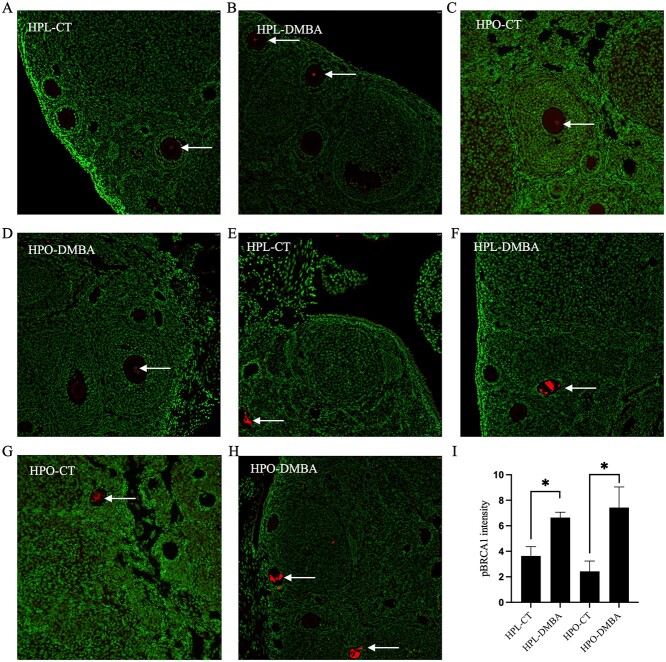
Impact of DMBA on oocyte pBRCA1 localization in lean and obese mice. Following 7 days of exposure to vehicle control (CT) or DMBA, immunostaining of pBRCA1 (red) was observed in oocyte nuclei of healthy follicles (indicated by arrows) in (A) HPL-CT, (B) HPL-DMBA, (C) HPO-CT, and (D) HPO-DMBA. Staining of pBRCA1 was also observed in atretic follicles (indicated by arrows) in (E) HPL-CT, (F) HPL-DMBA, (G) HPO-CT, and (H) HPO-DMBA and quantified in (I) (treatment effect *P* = 0.003; obesity effect *P* = 0.85; interaction effect *P* = 0.37). Green stain represents cellular DNA. Images were captured at 20× magnification. *n* = 4 ovaries from individual mice per treatment.

Localization of pBRCA1 was observed in the oocyte nuclei of healthy follicles (Figure 8A–D), and in atretic follicles (Figure 8E–H) in all treatment groups using immunofluorescent stains. Staining was consistent in the oocyte nuclei of larger follicles (secondary and pre-antral); however, not enough oocyte nuclei were available for quantification purposes and no observable treatment effects were noted. Positive pBRCA1 staining was not observed in the oocyte nuclei of primordial and primary follicles. In atretic follicles, the abundance of the pBRCA1 was increased due to DMBA exposure in lean and obese mice (Figure 7I; *P* < 0.05).

## Discussion

Originally associated with affluence, obesity currently affects all populations globally [32]. In females, obesity impairs fertility by reducing oocyte quality [43], decreasing fecundity [36], and increasing risks of birth defects [37] and is also associated with polycystic ovarian syndrome [44]. The percentage of obese adults in the United States is over 40% [45], and, since 1980, the prevalence of obesity has doubled in ~70 countries [32]. Among US adult women, higher rates of obesity are observed in minority populations with the most affected groups being non-Hispanic black (54.8%), followed by Hispanic (50.6) as compared to non-Hispanic white (38.0%) women [45]. The incidence of obesity worsened during the COVID-19 pandemic, with 48% of surveyed US adults gaining body weight in the first year of the pandemic [46], though it cannot be determined at this point if this weight gain resulted in obesity. Given the short time frame in which obesity has progressed into an epidemic, genetic composition accounts for only a small fraction of causes for this malady, but epigenetic alterations may have a greater role in promoting obesity [47]. In humans, children of obese parents are more prone to developing obesity [48]. In rodent models, maternal nutritional regulation causes epigenetic changes pertaining to growth factors and appetite control in the gametes of the offspring [49]. In mice, basal ovarian DNA damage is caused by obesity and a blunted response to genotoxicant exposure in obese compared to their lean counterparts has been demonstrated [20,21]. In this study, a hyperphagia-induced model of obesity was used where agouti lethal yellow mice naturally overeat due to a dominant mutation (A^vy^) in the pleiotropic agouti gene [50]. Obesity is a phenotypic consequence of the antagonistic effect of the agouti peptide to the hypothalamic melanocortin receptor [51,52]. The mice display hyperinsulinemia, hyperleptinemia [53,54], leptin resistance [54], and hyperphagia. The lethal yellow mice have reduced oocyte quality [55] and premature loss of fertility [56]. Previous work from our group has also discovered primordial follicle loss due to obesity in a time-dependent manner [38].

DMBA is a PAH chemical member, which are ovotoxicants liberated during combustion of organic matter [10,57]. Other sources of PAH exposures are from combustion of organic matter such as coal, oil, and wood [57]. Once absorbed, DMBA is biotransformed in the ovary to a genotoxic metabolite [18], resulting in DNA damage and increased abundance of DDR proteins [21]. The carcinogenic properties of DMBA are influenced by ovarian hormones and the ovarian cycle as ovariectomy drastically suppresses the incidence of DMBA-induced carcinogenesis in mammary glands [58]. Since ovarian toxicity of DMBA depends on its bioactivation by several enzymes including EPHX1 [18], it is difficult to estimate human DMBA ovarian exposure, given differential xenobiotic biotransformation between individuals [46]. DMBA exposure results in follicle loss at all stages, both in vivo [20] and in vitro [18,59], leading to permanent infertility. DSBs caused by DMBA exposure in the germline genome can cause genomic instability and pose a menace to the integrity of the germ cell. Furthermore, exposure to DMBA induces ovarian DDR [21] but the mechanism of repair in the female gamete remains to be fully elucidated. Exposure of lean and obese mice to the same dose of DMBA for 14 days caused almost complete destruction of primordial follicles and few primary and larger stage follicles remained in the ovary [20], precluding investigating mechanisms of primordial and primary follicle destruction due to the follicle stages of interest being almost completely absent. In this study, exposure to DMBA was at a dose demonstrated to be ovotoxic (1 mg/kg/day) but for 7 days rather than 14 days as in previous studies [20,21] to ensure that modes of ovotoxicity could be investigated in small pre-antral follicles that underwent exposure but which had not been depleted form the ovary.

Mice were 9 weeks of age at the beginning of the dosing period to ensure a 30% weight difference between the lean and obese groups, but more importantly, at a time point which is earlier than when obesity’s earliest effects on follicle numbers have been previously documented [38]. By experimental design, body weight was increased by hyperphagia at the time of tissue collection and this corresponded with increased feed intake by the obese group. High prevalence of nonalcoholic fatty liver disease has been associated with obesity [60] and higher weights of livers were observed in obese compared to lean females. Previously, DMBA exposure in adult mice for 2 weeks reduced ovarian weight in both lean and obese mice [20]; however, after 1 week exposure to the same DMBA dose, this effect was absent. Exposure to DMBA did decrease uterine weight in obese mice, which could be due to endocrine disruption or a direct effect of DMBA on uterine cellular viability.

Obesity is known to alter the duration spent at different phases of the estrous cycle in rodents. In mice, obesity reduces the duration of estrus and increases the duration of diestrus, starting at 18 weeks of age [38]. However, in the current experiment, in ~ 10-week-old mice, obesity did not alter the duration of time spent at each phase of the estrous cycle, in congruence with the estrous cyclicity data reported for ~9-week-old lean and obese mice [61]. In lean but not obese mice, DMBA exposure tended to increase the time spent at proestrus. There was no effect of DMBA exposure in lean or obese mice on circulating E2, though obese mice had lowered E2 when exposed to DMBA when compared to lean mice undergoing the same exposure. Thus, in this paradigm of obesity and DMBA exposure, there were few overt phenotypic signs of altered fertility in the mice, permitting investigation of initiating ovotoxic modes of action.

Given the large proportion of the general population affected by obesity, the impact on the ovarian reserve needs to be explored. In premenopausal women, the ovarian reserve is not reportedly affected by obesity [62–64]. However, our finding that obesity depletes primordial and primary follicle numbers [38] from 12 weeks of age onwards has since been recapitulated in rats [65]. Additionally, we have reported increased antral follicle number in obese mice [38]. At ~10 weeks of age, this trend was not observed in primordial follicles, but the number of primary and pre-antral follicles were reduced by obesity similar to our previous findings [38]. Reduced primary follicle number preceding a decline in primordial follicle number 2 weeks later [50] could reflect increased activation of primordial follicles from the ovarian reserve into the growing follicular pool, a mode of toxicity first observed during ovotoxicity caused by exposure to the ovotoxicant, 4-vinylcyclohexene diepoxide [66].

Exposure to DMBA tended to decrease primordial follicle number in lean but not obese mice suggesting that primordial follicle loss is induced before other follicle types by DMBA exposure, and that the experimental design was appropriate to study initiating modes of follicle destruction. In the obese mice, this decrease was ameliorated, potentially due to defective follicles being retained and obesity impairing the ability of the ovary to shunt the damaged follicles toward apoptosis. It is known that DDR molecules downstream of ATM are blunted due to DMBA exposure in obese mice, albeit in older mice [22]. It is also known that both ATM inhibition [67] and *Atm* haploinsufficiency [24] impair depletion of damaged ovarian follicles. Thus, this lack of primary follicle depletion by DMBA could support a defective ovarian DDR response. The number of preantral follicles was reduced due to obesity which is contradictory to obesity’s effects in older mice. This suggests that in this mouse model, the timeframe between 9 and 12 weeks of age is a window in which the ovary is able to react to damage and initiate apoptosis.

When the total ovarian abundance of the gold standard indicator of DSB [68,69], γH2AX, and the DDR repair protein BRCA1 were measured in the ovary, they were unaffected by either DMBA exposure or obesity. Epigenetic markers have been linked with ovarian dysfunction [70] and histone modifications identified to be involved in DNA repair—H3K4me [30], H4K5ac [31], H4K12ac [29,31], and H416ac [29]—were measured but no alterations in their total ovarian abundance in lean and obese mice exposed to DMBA were identified. In order to further explore any alteration to DNA repair in lean and obese mice exposed to DMBA, immunofluorescence staining was performed. In smaller follicles (primordial and primary), γH2AX staining was not visible but in antral follicles, there was a decrease in the number of γH2AX foci in obese mice as compared to lean. However, previously, an increase in γH2AX foci in small follicles has been reported in 10-week-old mice of the same mouse strain used herein [26]. This discrepancy could be due to smaller follicles in the previous study being designated as primary and secondary follicles combined. Staining of growing follicles only (secondary onwards) could indicate the retention of DNA damage in larger follicles at this time point. Although all stages of follicles have the capacity to repair DNA damage, primordial follicles are now known to efficiently respond to DNA damage and this time point was possibly too late to capture the DDR in action in smaller follicles [71]. When the expression of γH2AX in all follicles analyzed was combined, no difference was observed, consistent with the western blot data of the total abundance of γH2AX. Previous findings from our group have determined that in obese mice, there is a basal level of DNA damage as indicated by a total increase in γH2AX in older mice (18–20 weeks) [21,22] but in 10-week-old-obese mice, only a tendency for an increase in total γH2AX foci was observed via immunostaining and in this experiment, no increase in total γH2AX foci was noted. Conducting analysis of positively stained γH2AX foci throughout the entire ovary could be worthwhile in future studies.

In the oocyte, machinery for both homologous recombination (HR) and nonhomologous end joining is present, but DNA damage in primordial follicles is reportedly repaired by HR and it remains the predominant repair mechanism until metaphase II [72,73]. Initiation of the DDR in the oocytes was apparent when the distributions of the repair protein BRCA1 and its phosphorylated form were examined. BRCA1 is an essential member of the homology-directed DNA repair [74] and phosphorylation of BRCA1 by ATM can occur at several serine residues, the biological consequences of which are mostly unknown [75]; however, it is suggested that phosphorylation of BRCA1 at Ser-1524 regulates the localization of BRCA1 [76]. BRCA1 staining was evident on the oolemma of nearly all follicles in all treatment groups. Staining was also observed in the antral fluid, thecal cells, and atretic follicles. BRCA1 is a nuclear protein and is reportedly localized in the cytoplasm in breast and ovarian cancer cells [77]. Quantification of the intensity of the BRCA1 stain in oocytes revealed a decrease in BRCA1 in the oocytes of primary follicles in all treatment groups as compared with lean control. Previously our group has reported a decrease in BRCA1 abundance with progressive obesity [22] and in 10-week-old mice no observable changes in BRCA1 were noted suggesting that the alterations in BRCA1 previously noted could be age-related [24]. Herein decreased BRCA1 in the oocytes of primary follicles due to both DMBA and obesity exposure was observed. This study provides insight into the localization patterns of BRCA1 in the ovary. When the immunofluorescence signal from the oocytes of all follicles was combined, the intensity of BRCA1 was decreased due to DMBA exposure in lean mice but increased due to DMBA in obese mice. The contrasting trend suggests that obesity affects the cell’s ability to respond to DNA damage. It is important to note that this pattern is observed in the oocytes and while BRCA1 staining was observed in other parts of the ovary, it was not quantified in a cell-specific manner in other ovarian cells. In addition, quantification of the BRCA1 staining throughout the entire ovary would be a valuable approach. In order for BRCA1 to be involved in the DDR process, it needs to be phosphorylated [74] and the accumulation of total BRCA1 could potentially indicate a delay in its phosphorylation. This hypothesis needs to be supported by quantifying phosphorylated BRCA1 in the oocyte nuclei which was not possible due to the low number of oocyte nuclei stained by pBRCA1 in this experiment. Immunolocalization of pBRCA1 was observed in the oocyte nuclei in all treatment groups, and no apparent pattern was observed among treatment groups. The presence of basal DNA damage and the undergoing repair process was supported by the increased abundance of pBRCA1 due to DMBA exposure in the atretic follicles in both lean and obese mice. Whether BRCA1 is localized in the oocyte nucleus and migrates to the oolemma in an aberrant manner because of damage to the cell as seen in other cell populations, or it is localized in the oolemma and transported to the nucleus after phosphorylation to repair DNA damage needs further investigation.

In conclusion, this study indicates that there was tendency for reduced follicle number in lean mice which was absent in obese mice; thus, initiation of follicle loss is supported in lean mice exposed to DMBA and a differential lack of any impact in obese DMBA-treated mice. Additionally, lean mice had increased time spent in proestrus due to DMBA exposure and decreased BRCA1 intensity in primary follicles. Further, increased pBRCA1 staining was observed in atretic follicles of all DMBA-exposed mice. Thus, obesity can impair the ability of ovarian proteins with roles in the response to DNA damage to respond to DMBA exposure in a mouse model of hyperphagia-induced obesity. Although the total abundance of DNA repair proteins was not altered, immunolocalization of BRCA1 suggests a follicle-specific response which is altered in obese mice. Further investigation to understand the temporal mechanisms of the repair response by analyzing the localization patterns of pBRCA1 and other DDR proteins is warranted as well as examination of the temporal response to DMBA exposure to capture early onset molecular changes that facilitate efficient repair of damaged DNA.

## Data availability

Data available on request.

## Conflict of interest

The authors have declared that no conflict of interest exists.

## Authors’ contributions

AFK contributed to experimental conception and design. KT and HEW performed animal study and tissue collection. KCK provided intellectual and technical assistance with microscopy. JKR performed the experiments and data analysis. JKR drafted the paper. AFK reviewed, edited, and approved the final manuscript. 

## Supplementary Material

Supp_Figure_1_ioac218

Supp_Figure_2_ioac218
