# Supplementary figures and images for: Obesity partially potentiates dimethylbenz[a]anthracene-exposed ovotoxicity by altering the DNA damage repair response in mice
Source: Biol Reprod. 2023 Jan 25;108(4):694–707. doi: 10.1093/biolre/ioac218 (PMC10106840; doi:10.1093/biolre/ioac218)

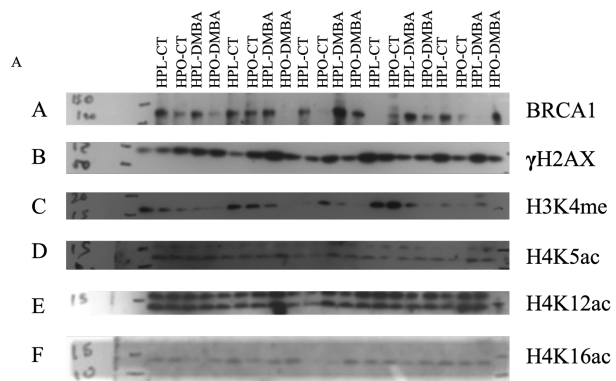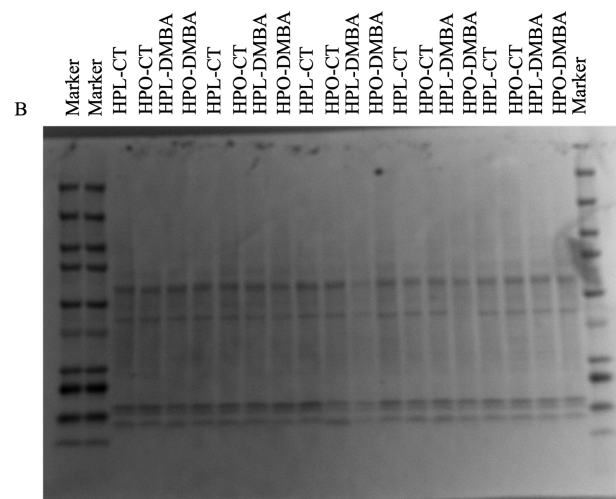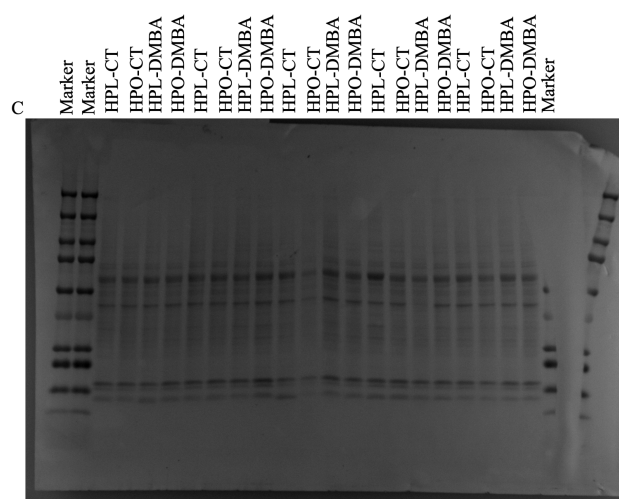

Supplement: Supp_Figure_1_ioac218 [file supp_figure_1_ioac218.pdf]

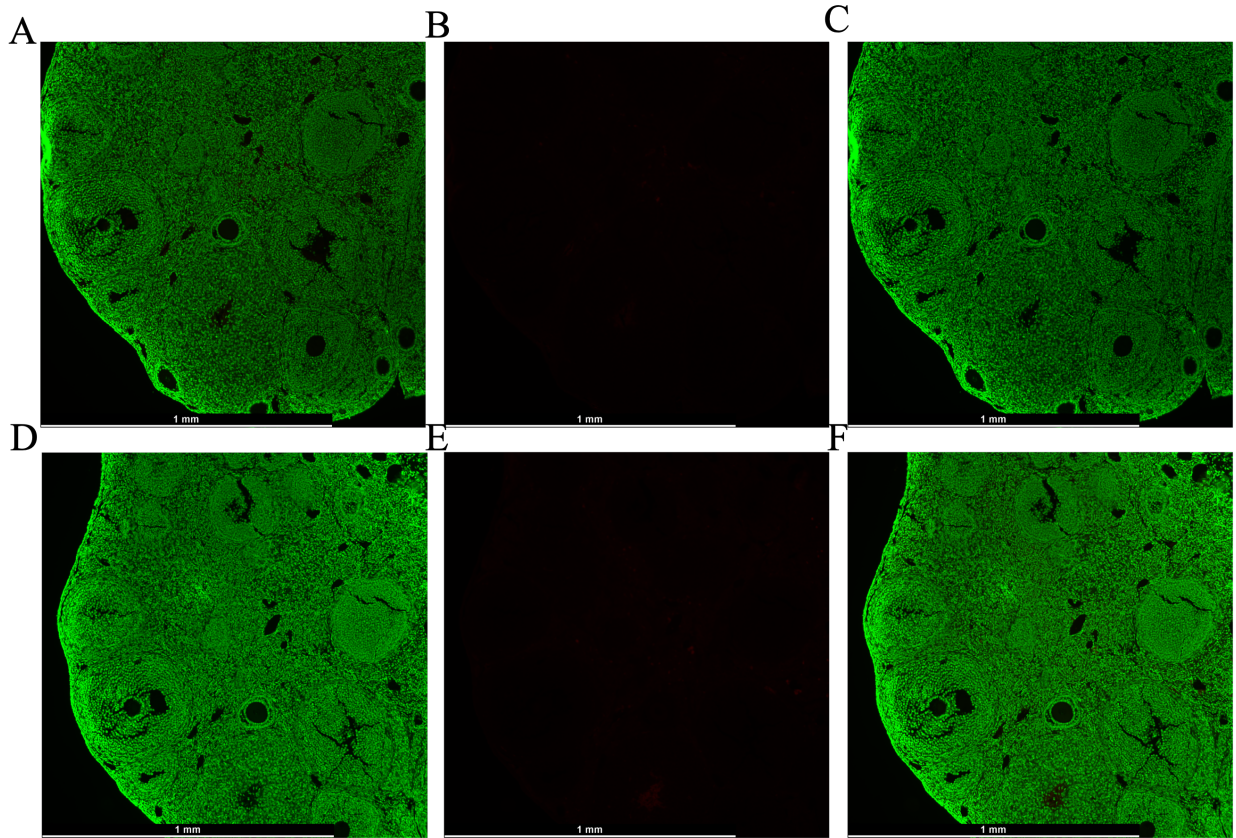

Supplement: Supp_Figure_2_ioac218 [file supp_figure_2_ioac218.pdf]
